# Associated Factors and Clinical Implication of Cutaneous Allodynia in Patients with Cluster Headache: A Prospective Multicentre Study

**DOI:** 10.1038/s41598-019-43065-1

**Published:** 2019-04-25

**Authors:** Byung-Su Kim, Jeong Wook Park, Jong-Hee Sohn, Mi Ji Lee, Byung-Kun Kim, Min Kyung Chu, Jin-Young Ahn, Yun-Ju Choi, Tae-Jin Song, Pil-Wook Chung, Kyungmi Oh, Kwang-Soo Lee, Soo-Kyoung Kim, Kwang-Yeol Park, Jae Myun Chung, Heui-Soo Moon, Chin-Sang Chung, Soo-Jin Cho

**Affiliations:** 1 Department of Neurology, Bundang Jesaeng General Hospital, Daejin Medical Center, Seongnam, South Korea; 2 Department of Neurology, Uijeongbu St Mary’s Hospital, The Catholic University of Korea, College of Medicine, Uijeongbu, South Korea; 3 Department of Neurology, Chuncheon Sacred Heart Hospital, Hallym University College of Medicine, Chuncheon, South Korea; 4 Department of Neurology, Neuroscience Center, Samsung Medical Center, Sungkyunkwan University School of Medicine, Seoul, South Korea; 5 Department of Neurology, Eulji Hospital, Eulji University, Seoul, South Korea; 6 Department of Neurology, Severance hospital, Seoul, South Korea; 7 Department of Neurology, Seoul Medical Center, Seoul, South Korea; 8 Department of Neurology, Presbyterian Medical Center, Jeonju, South Korea; 9 Department of Neurology, Ewha Womans University School of Medicine, Seoul, South Korea; 10 Department of Neurology, Kangbuk Samsung Hospital, Sungkyunkwan University School of Medicine, Seoul, South Korea; 11 Department of Neurology, Korea University College of Medicine, Seoul, South Korea; 12 Department of Neurology, Seoul St. Mary’s Hospital, Catholic University of Korea College of Medicine, Seoul, South Korea; 13 Department of Neurology, Gyeongsang National University College of Medicine, Jinju, South Korea; 14 Department of Neurology, Chung-Ang University Hospital, Seoul, South Korea; 15 Department of Neurology, Inje University College of Medicine, Seoul, South Korea; 16 Department of Neurology, Dongtan Sacred Heart Hospital, Hallym University College of Medicine, Hwaseong, South Korea

**Keywords:** Chronic pain, Migraine

## Abstract

Cutaneous allodynia (CA) is an abnormal pain in response to non-painful stimuli. In the present study, we sought to investigate the presence of CA, its associated factors, and its clinical implications in patients with cluster headache (CH). In this cross-sectional study, we analysed data from a prospective multicentre registry enrolling consecutive patients with CH. We identified CA during and between headache attacks using the 12-item Allodynia Symptom Checklist (ASC) administered during the CH bout period. Comorbid depression and anxiety were ascertained using the Patient Health Questionnaire (PHQ-9) and the Generalized Anxiety Disorder (GAD-7) scales. Headache impact was evaluated using the Headache Impact Test-6 (HIT-6). Of 119 eligible patients, 48 and two (40.3% and 1.7%) had CA during and between headache attacks, respectively. In univariable analyses, total CH duration, major depressive disorder (MDD), and generalized anxiety disorder (GAD) were associated with CA during headache attack. They remained significantly associated with CA during headache attack in multivariable analyses. Patients with CA during headache attack had higher headache impact (*P* = 0.002). A “50% responder” analysis showed no difference in outcome of acute and preventive treatment between patients with and without CA during headache attack. Patients with CH commonly experienced CA during headache attack, but not between headache attacks. CA during headache attack was associated with disease duration, depression, and anxiety.

## Introduction

Cutaneous allodynia (CA) is a clinical manifestation of pain or discomfort in response to non-painful stimuli that occurs in up to 80% of patients with migraine^1–4^. Over the past two decades, CA in patients with migraine has piqued increasing interest among researchers, and many studies have explored its pathophysiology and clinical implications. In particular, CA during a migraine attack is presently recognized as a surrogate marker of sensitization of central pain-signalling neurons in the trigeminal nucleus caudalis^5,6^. Furthermore, the symptom has been associated with triptans inefficacy, depression, and migraine chronification, suggesting that it has more associations than somatic pain alone^7–10^.

Cluster headache (CH) is a relatively rare primary headache disorder characterized by recurrent attacks of severe headache centred in the ophthalmic division of the trigeminal nerve. It involves ipsilateral cranial autonomic symptoms and has a relapsing–remitting course, with regular headache attacks occurring during the bout periods^11,12^. Despite some common points of pathophysiology, clinical manifestations, and treatment between migraine and CH, previous studies have shown inconsistent results with regard to the presence of CA in CH^13–18^. The first observation reported that 40% of CH patients were allodynic^13^, whereas quantitative sensory testing study found an opposite result, increased sensory thresholds to mechanical and thermal stimuli in CH patients^15^. In a recent largest study on this issue, 35.9% of patients with CH experienced CA during a headache attack, and CA was independently associated with depression^19^.

Regarding CA in CH, the present study sought to ascertain (1) the presence of CA among patients with CH in the Asian population, (2) the relation between CA and anxiety in patients with CH, (3) the association between CA severity and psychiatric comorbidities, and (4) the clinical implications of CA including headache impact and treatment response. Therefore, using a multicentre registry of patients with CH, we investigated the presence of CA during and between headache attacks, the factors associated with CA, the relationship between CA severity and psychiatric comorbidities, and the influence of CA on headache impact and treatment response.

## Methods

### Study design and patients

This was a prospective, cross-sectional study that used data from the Korean Cluster Headache Registry, which is a prospective, multicentre registry of consecutive patients with CH aged ≥19 years. The registry involves 15 hospitals across Korea—13 university hospitals (eight tertiary and five secondary referral) and two secondary referral general hospitals. The study protocol was approved by the local ethics committee or Institutional Review Board in each study hospital (Bundang Jesaeng General Hospital, Uijeongbu St.Mary’s Hospital, Chuncheon Sacred Heart Hospital, Samsung Medical Center, Eulji Hospital, Sacred Heart Hospital, Seoul Medical Center, Presbyterian Medical Center, Ewha Womans University Mokdong Hospital, Kangbuk Samsung Hospital, Korea University Kuro Hospital, Seoul St. Mary’s Hospital, Gyeongsang National University Hospital, Chung-Ang University Hospital, Seoul Paik Hospital, and Dongtan Sacred Heart Hospital) and conformed to the Declaration of Helsinki and Good Clinical Practice guidelines. All patients totally understood the study proposal and provided informed written consent before their participation.

We analysed the data of patients enrolled between September 2016 and February 2018. CH was diagnosed by experienced headache neurologists using the clinical criteria found in the 3rd edition of the International Classification of Headache Disorder, Beta Version (ICHD-3β)^20^. Since our main research subject was the presence of CA, we included patients who enrolled during the bout period of CH. The exclusion criteria were as follows: (1) enrolment during remission period, (2) possible secondary cause, (3) no or incomplete response to allodynia questionnaire, (4) missing clinical variables, and (5) lack of follow-up visits.

### Measurement

We used the 12-item Allodynia Symptom Checklist (ASC) to determine the presence of CA both during and between headache attacks^4^. Thus, the baseline questionnaire consisted of two ASCs. The item responses for each allodynia symptom were categorized as follows: “never,” “rarely,” “less than half the time,” and “half the time or more.” These responses were converted into scores as follows: 0 (never, rarely, or does not apply to me), 1 (less than half the time), and 2 (half the time or more). Thus, the total ASC score ranged from 0 to 24. Based this score, the patients were classified into four allodynia severity groups: no CA (0–2), mild CA (3–5), moderate CA (6–8), and severe CA (9–24)^4^. In terms of the presence of CA, the study patients were dichotomously divided into a no CA (ASC score ≤2) group and a CA (ASC score ≥3) group.

We used the following clinical information in our analysis: demographic factors, social habits, headache characteristics, psychiatric comorbidity, headache impact, and treatment response. Psychiatric comorbidity was assessed using the Korean versions of the Patient Health Questionnaire 9-item scale (PHQ-9) and the Generalized Anxiety Disorder 7-item scale (GAD-7). Major depressive disorder (MDD) and generalized anxiety disorder (GAD) were identified in patients with PHQ-9 and GAD-7 scores of ≥10, respectively^21^. If patients had either MDD or GAD, they were considered as having a psychiatric comorbidity. Headache impact was evaluated using the Headache Impact Test-6 (HIT-6)^22^. The appropriate CH treatment was determined individually by each investigator, and treatment response was investigated at the follow-up visits. Treatment-response assessment of study patients was conducted during their follow-up reservations as a part of daily clinical practice. Study patients usually started both acute and preventive therapy right after the enrolment. Given action time of some preventive treatment (verapamil or lithium), we set the judgement point at least 2 weeks after an initial visit. Treatment outcome was assessed using analysis of 50% responders, defined as any patient who showed ≥50% relief of headache attacks after acute treatment, or a ≥50% reduction in headache frequency after preventive treatment.

### Statistical analysis

Continuous variables are shown as means ± standard deviation, while categorical variables are given as numbers (percentage). The chi-square, Fisher’s exact, or linear-by-linear association test was used to compare categorical variables. To identify factors associated with CA, we conducted logistic regression analyses. The results of the univariable analyses are given odds ratios (ORs) and 95% confidence intervals (CIs). Because age, female sex, comorbid migraine may be associated with allodynia, the multivariate models were adjusted for these variables, regardless of whether they showed statistical significance in the univariable analyses^8,19^. All factors with P-values < 0.05 in the univariable analyses were considered potential covariates and were entered into the multivariable model. If any pair of potential covariates were closely correlated, we conducted separate multivariable models. Independent CA variables were presented as multivariable-adjusted ORs (aORs) and 95% CIs. All statistical analyses were conducted using SPSS for Windows (version 18.0; IBM Corp., Armonk, NY, USA). All reported *P*-values are two-tailed, and those <0.05 were considered statistically significant.

## Results

### Study patients

During the study period, 159 patients were prospectively enrolled in the registry. Forty patients were excluded because they had one or more of the exclusion criteria: enrolment during a remission period (n = 40), possible secondary cause (n = 2), no or incomplete response to allodynia questionnaire (n = 21), and missing clinical variables (n = 7). Therefore, we conducted the first analysis using data from 119 eligible patients. Next, we performed a treatment-response analysis after excluding seven patients who failed to show up to their follow-up reservations. With regards to headache classification, 101 patients (84.8%) definitely fulfilled the clinical criteria for first cluster period of CH (ICHD-3β code 3.1), while 18 patients (15.2%) were diagnosed as having probable CH (ICHD-3β code 3.5.1) and five (4.2%) were classified as having chronic CH. The mean age at enrolment was 38.5 ± 11.3 years, while that of CH onset was 30.1 ± 13.3 years, respectively (Table 1). Since 20 patients (16.8%) were women, the male-to-female ratio was 4.95 to 1. Eighteen patients had a coexisting history of migraine (15.1%). Baseline characteristics of age, female sex, coexisting migraine history, total duration of CH illness, duration of cluster headache bout, attack duration, MDD, and GAD in patients who completed the ASC did not significantly differ from those of patients excluded due to incomplete response to the ASC.

**Table 1:** Baseline characteristics and univariable analysis on factors associated with cutaneous allodynia during headache attack.

	Total (n = 119)	CA (n = 48)	No CA (n = 71)	OR (95% CI)	*P*
Age, years	38.5 ± 11.3	39.2 ± 10.4	38.0 ± 11.8	1.00 (0.97–1.04)	0.588
Female sex, no. (%)	20 (16.8)	11 (22.9)	9 (12.7)	2.04 (0.77–5.40)	0.148
Body mass index, kg/m^2^	23.9 ± 3.1	23.8 ± 3.2	24.0 ± 3.0	0.97 (0.86–1.09)	0.687
Current smoking, no. (%)	54 (45.4)	25 (52.1)	29 (40.8)	1.57 (0.75–3.29)	0.228
Alcohol drinking, no. (%)	61 (51.3)	25 (52.1)	36 (50.7)	1.05 (0.50–2.19)	0.883
Coexisting migraine history, no. (%)	18 (15.1)	8 (16.7)	10 (14.1)	1.22 (0.44–3.35)	0.700
Onset age of cluster headache, years	30.1 ± 13.3	28.0 ± 13.2	31.5 ± 13.3	0.97 (0.95–1.00)	0.161
Total duration of CH illness, years	8.4 ± 8.1	11.2 ± 9.3	6.5 ± 6.7	1.07 (1.02–1.13)	0.003
Recurrent bout, no. (%)	96 (80.6)	39 (81.3)	57 (80.3)	1.06 (0.41–2.70)	0.896
Duration of cluster headache bout, weeks	10.3 ± 38.9	17.1 ± 60.1	5.8 ± 8.8	1.01 (0.99–1.03)	0.244
Attack frequency per day	2.2 ± 1.9	2.5 ± 2.4	2.0 ± 1.4	1.13 (0.93–1.36)	0.199
Attack duration, minutes	93.1 ± 62.8	89.5 ± 52.0	95.6 ± 69.4	0.99 (0.99–1.00)	0.602
Headache intensity (0–10 VAS)	8.9 ± 1.3	9.0 ± 1.2	8.9 ± 1.3	1.08 (0.81–1.45)	0.565
MDD, no. (%)	37 (31.1)	21 (43.8)	16 (22.5)	2.67 (1.20–5.93)	0.016
GAD, no. (%)	44 (37.0)	26 (54.2)	18 (25.4)	3.48 (1.59–7.59)	0.002
Psychiatric comorbidity (either MDD or GAD), no. (%)	55 (46.2)	31 (64.6)	24 (33.8)	3.57 (1.65–7.70)	0.001

The data are shown as the mean ± standard deviation or number (percentage).

Abbreviations: CA, cutaneous allodynia; CH, cluster headache; CI, confidence interval; GAD, generalized anxiety disorder; MDD, major depressive disorder; OR, odds ratio; VAS, visual analogue scale.

### Presence of cutaneous allodynia in patients with cluster headache

**Figure 1:**
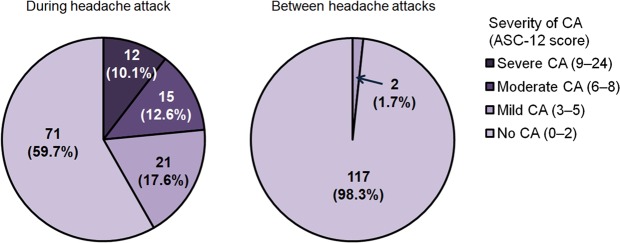
Presence of cutaneous allodynia during and between headache attacks in patients with cluster headache. Abbreviations: ASC, Allodynia Symptom Checklist; CA, cutaneous allodynia.

The analysis of the presence of CA is summarized in Fig. 1. During headache attack, 40 (40.3%) patients had CA, while 22.7% experienced moderate-to-severe CA. Between headache attacks, mild CA was reported in two patients (1.7%).

### Factors associated with cutaneous allodynia during headache attack

Factors that may be associated with CA during headache attack were identified by applying demographics, clinical characteristics of CH, and psychiatric comorbidities (Table 1). Univariable analyses revealed that the total CH duration was significantly associated with CA during headache attack (OR: 1.07, 95% CI: 1.02–1.13). Headache attack frequency, attack duration, pain intensity, and duration of CH bout were not related to CA during headache attack. With regards to psychological aspects, comorbid MDD and GAD were significantly associated with CA during headache attack (OR: 2.67, 95% CI: 1.20–5.93 and OR: 3.48, 95% CI: 1.59–7.59, respectively).

In the multivariable models, the ORs of total duration of CH illness were significantly increased (OR: 1.08, 95% CI: 1.02–1.13 in model 1; OR: 1.06, 95% CI: 1.01–1.12 in model 2; OR: 1.07, 95% CI: 1.01–1.13 in model 3; Table 2). MDD, GAD, and psychiatric comorbidity were also found to be significantly associated with CA during headache attack (OR: 2.37, 95% CI: 1.01–5.55 for MDD; OR: 2.74, 95% CI: 1.20–6.28 for GAD; OR: 2.91, 95% CI: 1.30–6.52 for psychiatric comorbidity).

**Table 2:** Independent variables of cutaneous allodynia during headache attack.

	Model 1		Model 2		Model 3	
	aOR (95% CI)	*P*	aOR (95% CI)	*P*	aOR (95% CI)	*P*
Age	1.00 (0.96–1.03)	0.886	1.00 (0.97–1.04)	0.745	1.00 (0.96–1.04)	0.842
Female sex	2.11 (0.72–6.13)	0.168	2.45 (0.84–7.17)	0.100	2.26 (0.77–6.60)	0.133
Coexisting migraine history	1.24 (0.40–3.82)	0.700	1.26 (0.41–3.85)	0.675	1.33 (0.43–4.09)	0.612
Total duration of CH illness (per year)	1.08 (1.02–1.13)	0.003	1.06 (1.01–1.12)	0.014	1.07 (1.01–1.13)	0.010
MDD	2.37 (1.01–5.55)	0.046				
GAD			2.74 (1.20–6.28)	0.017		
Psychiatric comorbidity (either MDD or GAD)					2.91 (1.30–6.52)	0.009

Age, female sex, coexisting migraine history, and potential variables (*P* < 0.10 at univariable analyses) were selected to develop multivariable models.

MDD, GAD, and psychiatric comorbidity were entered into different models, because they were correlated each other.

Abbreviations: aOR, multivariable-adjusted odds ratio; CH, cluster headache; CI, confidence interval; GAD, generalized anxiety disorder; MDD, major depressive disorder.

Association between stratum of total duration of CH illness and CA during headache attack was further examined in univariable and multivariable analyses (Table 3). Compared to total duration of CH illness ≤1 year, total duration of CH illness >10 years was associated with CA during headache attack in univariable analysis (OR: 2.66, 95% CI: 1.01–7.03). The association tended to increase across the stratum of total duration of CH illness (*P* for trend = 0.019). The association remained significant after adjustment for potential covariates.

**Table 3:** Association of total duration of cluster headache illness with cutaneous allodynia during headache attack.

	Univariable analysis		Model 1		Model 2		Model 3	
	OR (95% CI)	*P*	aOR (95% CI)	*P*	aOR (95% CI)	*P*	aOR (95% CI)	*P*
Stratum of total duration of CH illness								
≤1 year	reference		reference		reference		reference	
1< and ≤5 years	0.89 (0.29–2.73)	0.843	1.05 (0.31–3.50)	0.935	0.92 (0.27–3.10)	0.901	1.11 (0.32–3.75)	0.865
5< and ≤10 years	1.33 (0.43–4.08)	0.608	1.89 (0.57–6.28)	0.294	1.67 (0.50–5.56)	0.403	1.85 (0.55–6.21)	0.316
>10 years	2.66 (1.01–7.03)	0.048	3.75 (1.31–10.73)	0.013	2.90 (1.01–8.30)	0.047	3.34 (1.17–9.58)	0.024
*P* for trend		0.019		0.005		0.019		0.011

Model 1: adjustment for age, female sex, coexisting migraine history, and MDD; Model 2: adjustment for age, female sex, coexisting migraine history, and GAD; Model 3: adjustment for age, female sex, coexisting migraine history, and psychiatric comorbidity (either MDD or GAD).

Abbreviations: aOR, multivariable-adjusted odds ratio; CH, cluster headache; CI, confidence interval; GAD, generalized anxiety disorder; MDD, major depressive disorder; OR, odds ratio.

**Figure 2:**
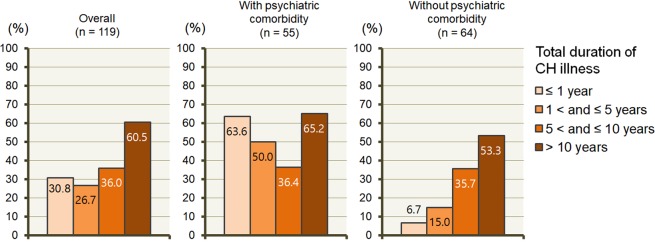
Proportions of patients with cutaneous allodynia during headache attack according to total duration of cluster headache illness. Abbreviation: CH, cluster headache.

The relationship between total duration of CH illness and CA was analysed by dividing the patients into two groups according to the presence of psychiatric comorbidity (Fig. 2). The proportion of CA during headache attack was increased according to the category of total duration of CH illness: 30.8% after ≤1 year, 26.7% between 1 and ≤5 years, 36.0% between 5 and ≤10 years, and 60.5% after 10 years; *P* = 0.019. In patients without psychiatric comorbidity, there was a clear dose–response relationship between CA during headache attack and total duration of CH illness: 6.7% after ≤1 year, 15.0% between 1 and ≤5 years, 35.7% between 5 and ≤10 years, and 53.3% after 10 years; *P* = 0.013.

### Association between cutaneous allodynia severity during headache attack and psychiatric comorbidity

**Figure 3:**
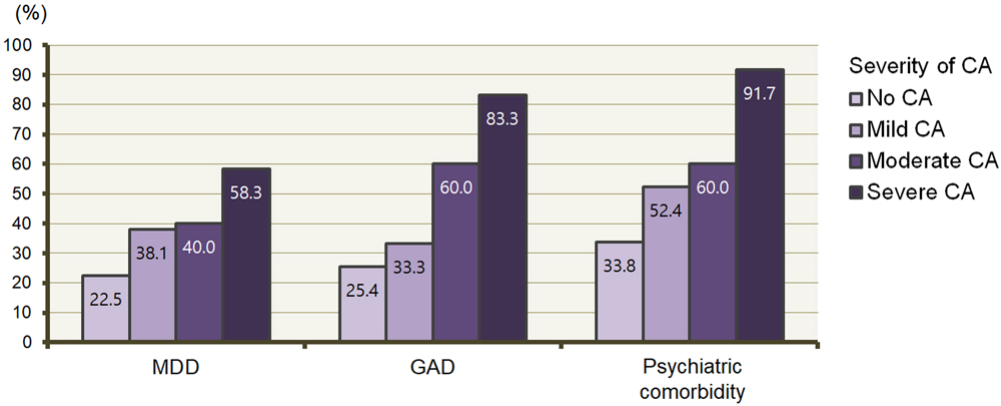
Proportions of patients with major depressive disorder, generalized anxiety disorder, and psychiatric comorbidity according to severity of cutaneous allodynia during headache attack. Abbreviations: CA, cutaneous allodynia; GAD, generalized anxiety disorder; MDD, major depressive disorder.

The proportions of patients with MDD, GAD, and psychiatric comorbidity were compared in terms of CA severity during headache attack (Fig. 3). The prevalences of MDD, GAD, and psychiatric comorbidity were highest in patients with severe CA during headache attack (58.3%, 83.3%, and 91.7%, respectively). The prevalence of MDD increased with the CA severity during headache attack, although not significantly (*P* = 0.055). The prevalences of GAD and psychiatric comorbidities increased significantly with the CA severity during headache attack (*P* < 0.001 and *P* = 0.001, respectively).

### Cutaneous allodynia during headache attack and headache impact

**Figure 4:**
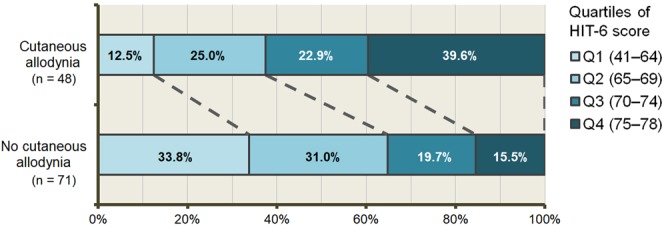
Headache impact between patients with and without cutaneous allodynia during headache attack. Abbreviations: HIT-6, Headache Impact Test-6; Q1, 1st quartile; Q2, 2nd quartile; Q3, 3rd quartile; Q4, 4th quartile.

The quartiles of the HIT-6 scores were compared in terms of presence of CA during headache attack (Fig. 4). The prevalences of 3rd- and 4th-quartile HIT-6 scores were significantly higher in patients with CA during headache attack sthan in those without (22.9% and 39.6% vs. 19.7% and 15.5%, respectively; *P* = 0.002).

### Cutaneous allodynia during headache attack and treatment response

The 50% responder analysis is summarized in Table 4. The number of patients receiving each CH treatment was as follows: oxygen (n = 13), NSAIDs (n = 17), combined analgesics (n = 11), triptans (n = 78), oral corticosteroid (n = 53), occipital nerve block (n = 9), verapamil (n = 47), and lithium (n = 16). The treatment response did not differ significantly between patients with and without CA during headache attack in any of the CH treatment subgroups.

**Table 4:** Treatment response analysis: numbers and proportions of 50% percent responder to cluster headache treatment between patients with and without cutaneous allodynia during headache attack.

	CA (n = 48)	No CA (n = 71)	P*
Acute treatment			
Oxygen	7/7 (100.0)	3/6 (50.0)	0.070
NSAIDs	6/6 (100.0)	10/11 (90.9)	1.000
Combined analgesics	4/7 (57.1)	2/4 (50.0)	1.000
Triptans	36/37 (97.3)	36/41 (87.8)	0.204
Preventive treatment			
Oral corticosteroid	26/29 (89.7)	23/24 (95.8)	0.617
Occipital nerve block	3/4 (75.0)	4/5 (80.0)	1.000
Verapamil	22/24 (91.7)	18/23 (78.3)	0.245
Lithum	8/10 (80.0)	3/6 (50.0)	0.299

The data are shown as the number (percentage).

*Comparison of the proportions of 50% percent responder between patients with and without cutaneous allodynia during headache attack.

Abbreviations: CA, cutaneous allodynia; NSAIDs, nonsteroidal anti-inflammatory drugs.

## Discussion

In the present prospective study, CA during headache attack was present in 40.3% of patients with CH, whereas only 1.7% experienced CA between headache attacks. Total duration of CH illness, MDD, and GAD were independently associated with CA during headache attack. Patients without psychiatric comorbidity showed a marked association between disease duration and CA during headache attack, and there was a dose–response relationship between CA severity during headache attack and psychiatric comorbidity. Patients with CA during headache attack suffered from greater headache impact. However, there was no significant difference in treatment response between patients with and without CA during headache attack.

In line with the Dutch study, the results of the current study indicate that CA during headache attack occurs in about 40% of patients with CH^19^. This consistency of results is interesting because the two study populations differ greatly in terms of ethnicity and sociocultural environment. It follows that the presence of CA in CH is likely relatively consistent among different populations. In addition, it is important to note that both studies used the ASC-12 to evaluate the presence of CA, rather than quantitative sensory testing, which is the gold standard for determining allodynia. The ASC-12 has not been comprehensively validated in patients with CH, while quantitative sensory testing has. Nevertheless, the Dutch study reported an acceptable outcome in terms of external validation of the ASC-12^19^. Given that our results were analogous to those of the Dutch study, it is likely that the ASC-12 questionnaire could be used to determine the presence of CA in future studies on this topic. The current study had an additional strength: we analysed the data of patients who responded to the questionnaire during a CH bout. This may have minimized the risk of recall bias in the questionnaire evaluation process.

With respect to the presence of CA between headache attacks (interictal CA), previous results have been inconsistent^13,14,16,18^. In the present study, only two patients (1.7%) experienced mild CA between headache attacks. The same patients had moderate-to-severe CA during headache attack, and their headache subtype was episodic CH. Neither had any coexisting migraine history or psychiatric comorbidity. These findings indicate that the major form of CA in patients with CH is acute allodynia during headache attack. Further studies are required to confirm our findings.

In the current study, we confirmed that CA is associated with both depression and anxiety in patients with CH, and that the frequencies of depression and anxiety increased with CA severity, partly corroborating the results of a population study of individuals who suffer migraines^8^. Notably, more than 90% of patients with severe CA had either depression or anxiety, suggesting that clinicians must consider psychological aspects in the management of patients with CH patients with CA. CA has been associated with comorbid affective disorders, perhaps because the conditions share a neuroanatomical site. In this regard, the prefrontal cortex and anterior cingulate cortex are thought to be the functional areas of the affective component of pain perception^23^. In particular, a previous functional MRI study showed that these areas were activated in response to virtual pain stimulation in patients with allodynia^24^. Furthermore, the substantia nigra pars compacta and ventral tegmental area are known as the action sites of multiple antidepressants. The trigeminal nucleus caudalis is adjacent to this site, which may explain the association between CA and depression^8^.

Previous studies have reported that cluster pain and ictal CA disappear in response to oxygen or triptans^13,16,18^. These results are somewhat contrary our expectation that CH patients with CA may have a lack of efficacy of acute treatment. In the present study, the presence of CA during headache attack did not significantly influence the outcome of acute or preventive therapy in patients with CH. Most patients were given preventive therapy at the same time treatment response was assessed, since they usually started both acute and preventive therapy after the enrolment. Therefore, when interpreting our results, we must remember that preventive therapy can affect the response to acute therapy and decrease allodynia^25^. Given that our assessment was a simple observation of daily clinical practice, the exact role of CA in CH treatment must be examined further in future investigations with an interventional design.

Previous investigations have been contradictory regarding the association between disease duration and allodynia in CH^13,14,17,19^. However, in the present study, longer disease duration was clearly associated with the presence of CA during headache attack, and the multivariable models consistently demonstrated that disease duration was significantly associated with ictal CA. Patients suffered from CH for >10 years, compared to those for ≤1 year, were at the risk of having ictal CA (OR: 3.34, 95% CI: 1.17–9.58 in multivariable model 3). These results suggest that CA in patients with CH is a clinical consequence of time-dependent processes in central pain signalling neuron^13,14^. Interestingly, this association became more noticeable in patients without psychiatric comorbidities. Meanwhile, the prevalence of CA during headache attack was ≥60% in patients with psychiatric comorbidities, even when the disease duration was short ≤1 year. Furthermore, CA tended to be more prevalent in patients with psychiatric comorbidity than in those without (56.3% vs. 26.5%). To summarise, allodynia in patients with CH may have a more intricate underlying mechanism involving central trigeminovascular neurons and higher cortical centres related to affective function and pain perception, as shown in previous neuroimaging studies^23,24,26–28^.

There were some limitations to our study. Firstly, since the data were based on a multicentre, hospital-based registry, there may be a risk of selection bias. However, given the prevalence of CH in population studies (0.1–0.3%), multicentre enrolment may be the optimal design to reduce the risk^11,12^. Secondly, the study may have lacked statistical power because the sample size was modest. Thirdly, the study enrolled only Korean patients. There is considerable dissimilarity between Western and Asian populations with CH^29–31^. Our results should be interpreted with this in mind. Fourthly, the proportion of patients with chronic CH was only 4.2% (n = 5) in our data. Of these, three patients had CA during headache attack, which was not significantly higher than in patients with episodic CH (60.0% vs. 39.5%; *P* = 0.392). Consequently, it was impossible to explore the relationship between CH subtype and CA in our study. Fifthly, despite the fact that we found a significant association between disease duration and ictal allodynia, total clustered periods or accumulated burden of cluster headache attacks might be other explanatory variables. However, we could not ascertain the association of those variables with ictal CA, due to a lack of data, which should be investigated in future studies. Finally, we used the ICHD-3β in the current study. There were some changes in the clinical criteria for CH between the ICHD-3β and the ICHD-3 final version. In this regard, the use of the ICHD-3β version may have led to some discrepancies^32^.

## Conclusions

We can confirm that a significant proportion of CH patients experience CA during headache attack, but rarely between headache attacks. The presence of CA was associated with disease duration and psychiatric comorbidity in patients with CH.
